# Ultramicronized palmitoylethanolamide rescues learning and memory impairments in a triple transgenic mouse model of Alzheimer’s disease by exerting anti-inflammatory and neuroprotective effects

**DOI:** 10.1038/s41398-017-0076-4

**Published:** 2018-01-31

**Authors:** Caterina Scuderi, Maria Rosanna Bronzuoli, Roberta Facchinetti, Lorenzo Pace, Luca Ferraro, Kevin Donald Broad, Gaetano Serviddio, Francesco Bellanti, Gianmauro Palombelli, Giulia Carpinelli, Rossella Canese, Silvana Gaetani, Luca Steardo, Luca Steardo, Tommaso Cassano

**Affiliations:** 1 Department of Physiology and Pharmacology “V. Erspamer”, SAPIENZA University of Rome, Rome, Italy; 2 Department of Clinical and Experimental Medicine, University of Foggia, Foggia, Italy; 3 Department of Life Sciences and Biotechnology, University of Ferrara, Ferrara, Italy; 4 UCL Institute of Opthalmology, University College, University College London, London, UK; 5 C.U.R.E. Centre for Liver Diseases Research and Treatment, Institute of Internal Medicine, Department of Medical and Surgical Sciences, University of Foggia, Foggia, Italy; 6 Department of Cell Biology and Neurosciences, Istituto Superiore di Sanità, Rome, Italy; 7 Department of Psychiatry, University of Naples SUN, Naples, Italy

**Keywords:** Molecular neuroscience, Learning and memory

## Abstract

In an aging society, Alzheimer’s disease (AD) exerts an increasingly serious health and economic burden. Current treatments provide inadequate symptomatic relief as several distinct pathological processes are thought to underlie the decline of cognitive and neural function seen in AD. This suggests that the efficacy of treatment requires a multitargeted approach. In this context, palmitoylethanolamide (PEA) provides a novel potential adjunct therapy that can be incorporated into a multitargeted treatment strategy. We used young (6-month-old) and adult (12-month-old) 3×Tg-AD mice that received ultramicronized PEA (um-PEA) for 3 months via a subcutaneous delivery system. Mice were tested with a range of cognitive and noncognitive tasks, scanned with magnetic resonance imaging/magnetic resonance spectroscopy (MRI/MRS), and neurochemical release was assessed by microdialysis. Potential neuropathological mechanisms were assessed postmortem by western blot, reverse transcription–polymerase chain reaction (RT-PCR), and immunofluorescence. Our data demonstrate that um-PEA improves learning and memory, and ameliorates both the depressive and anhedonia-like phenotype of 3×Tg-AD mice. Moreover, it reduces Aβ formation, the phosphorylation of tau proteins, and promotes neuronal survival in the CA1 subregion of the hippocampus. Finally, um-PEA normalizes astrocytic function, rebalances glutamatergic transmission, and restrains neuroinflammation. The efficacy of um-PEA is particularly potent in younger mice, suggesting its potential as an early treatment. These data demonstrate that um-PEA is a novel and effective promising treatment for AD with the potential to be integrated into a multitargeted treatment strategy in combination with other drugs. Um-PEA is already registered for human use. This, in combination with our data, suggests the potential to rapidly proceed to clinical use.

## Introduction

Alzheimer’s disease (AD) is the primary cause of dementia in the elderly, but currently prescribed medications provide only modest and transient benefits to a subset of patients.

Histopathologically, the major features of AD include the extracellular accumulation of beta amyloid (Aβ) fibrils in senile plaques (SPs) and intraneuronal neurofibrillary tangles (NFTs), whose precise role in the progression of AD remains to be clarified^1,2^. Interestingly, preclinical and clinical data have demonstrated that both SPs and NFTs are colocalized close to activated glial cells, suggesting that a dysfunction in glia homeostasis is a key pathogenetic mechanism in AD^3–5^. In the context of AD, both astrocytes and microglia can be activated by Aβ which promotes further reactive gliosis^6^. This phenomenon is normally engaged with the intent of defending the brain by removing injurious stimuli (e.g., Aβ fibrils phagocytosis). However, if prolonged, this response exceeds normal physiological limits and can induce detrimental effects^7–10^.

Our hypothesis is that an early combination of neuroprotective and anti-inflammatory treatments represents a promising treatment approach for the treatment of AD. The endogenous lipid mediator palmitoylethanolamide (PEA) demonstrates exceptional potential as a novel treatment for AD. We have previously demonstrated PEA anti-inflammatory and neuroprotective properties, as well as its ability to preserve memory function in rodent models of AD^11–16^. At present, we lack precise information concerning both the effects of chronic PEA administration on the progression of AD and the optimal time to begin treatment. This is an important consideration as one of the major problems with the development of effective treatments for AD is that diagnosis is normally made at an advanced stage of the disease which may mean many therapeutic interventions begin too late to be effective.

In this paper, we evaluated the effects of chronic um-PEA administration in 3×Tg-AD mice at two different stages (mild and severe) of AD-like pathology and cognitive deficits, by subcutaneously administering the drug to two age groups of animals for 3 months. 3×Tg-AD mice were chosen because they present both Aβ deposits and tau pathology, as well as synaptic dysfunction, thus representing a widely used and validated model which closely mimics the neuropathological alterations seen in human AD^17,18^. The animals were then tested using a range of cognitive and noncognitive tasks, followed by an assessment of neuropathology.

Our data demonstrate the first in vivo evidence that chronic treatment with ultramicronized-PEA (um-PEA), a formulation which maximizes its bioavailability^19,20^, induces considerable improvements in cognitive and neural function during both the early presymptomatic and later symptomatic stages of AD in a triple transgenic mouse model of AD (3×Tg-AD mice).

Our data suggest that PEA demonstrates exceptional potential as a novel treatment for AD and in combination with the fact that is already licensed for the use in humans, where it demonstrates high safety and tolerability, provides an opportunity for its rapid translation in clinical pactice.

## Materials and methods

### Animals and pellet implantation

3×Tg-AD (harboring APP_swe_, PS1_M146V_, and tau_P301L_ transgenes) male mice and their sex- and age-matched wild-type littermates (Non-Tg) (C57BL6/129SvJ) were maintained in controlled conditions (12-h light/12-h dark cycle, temperature 22 °C, humidity 50–60%, fresh food, and water ad libitum). All procedures were conducted in accordance with the guidelines of the Italian Ministry of Health (D.L. 26/2014) and the European Parliamentary directive 2010/63/EU.

Mice of 3 and 9 months were anesthetized by i.p. injection of ketamine hydrochloride (1 mg/10 g) and xylazine (0.1 mg/10 g). The area between the shoulder blades was shaved and the surgical area was sterilized with alcohol. A small (1–2 cm) dorsal midline incision was made and a subcutaneous pocket was created with a blunt probe. An um-PEA or a placebo pellet was placed into the pocket and the incision was closed with sterile sutures.

### Drugs and protocols

**Fig. 1:**
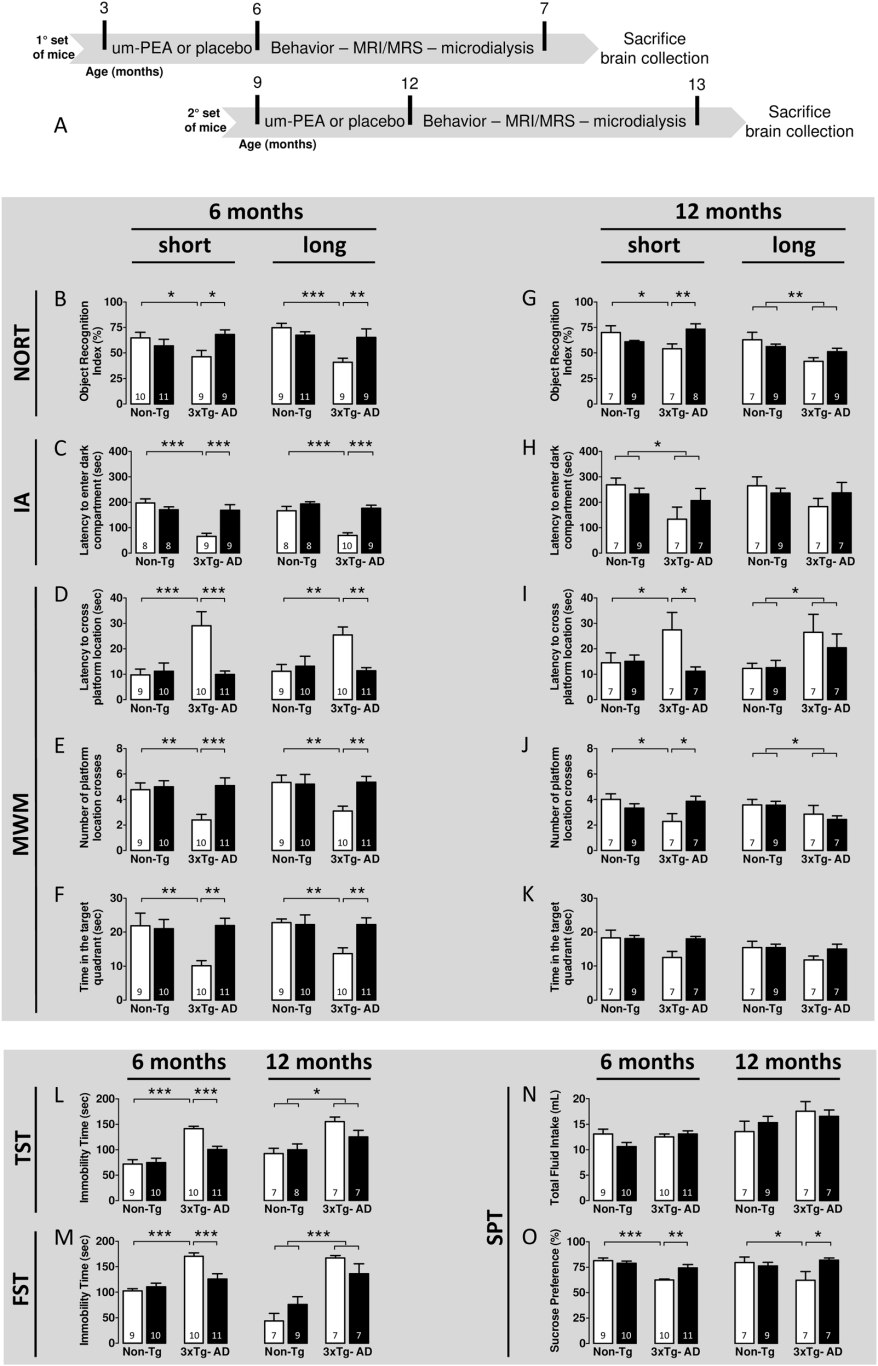
Um-PEA rescues early memory deficits and ameliorates the depressive-like phenotype in the 3×Tg-AD mice. (**a**) Schematic representation of the experimental design. Evaluation of the (**b–k**) cognitive and (**l–o**) emotional phenotype of 6- and 12-month-old 3×Tg-AD and age-matched Non-Tg mice chronically treated with placebo (open bars) or um-PEA (black bars). Short- and long-term memory of mice was evaluated by (**b**, **g**) novel object recognition test (NORT), (**c**, **h**) inhibitory passive avoidance (IA), and (**d**–**f**, **i**–**k**) Morris water maze (MWM). Moreover, the emotional phenotype of mice was evaluated by (**l**) tail suspension test (TST), (**m**) forced swim test (FST), and (**n**, **o**) sucrose preference test (SPT). Sample size is indicated in the bars. The data are presented as means ± SEM. Statistical analysis was performed by two-way ANOVA followed by Tukey multiple- comparison test (*p < 0.05; **p < 0.01; ***p < 0.001)

Um-PEA (EPT2110/1) was obtained from Epitech group (Saccolongo, Italy). A 90-day-release pellet containing either 28 mg of um-PEA (Innovative Research of America, Sarasota, Florida; cat. #NX-999) or placebo (cat. #NC-111) was subcutaneously implanted. During pellet inclusion process, um-PEA was homogeneously distributed in the matrix, maintaining its original crystalline form and micrometric size; both dosage and administration route were chosen according to previous data^20,21^. Both Non-Tg and 3 × Tg-AD mice were randomly assigned to either placebo or um-PEA group. No animals were excluded from the analysis. Behavioral sample size (N) is specified in open and black bars of Fig. 1. For molecular analyses, the sample size (N) for all experimental groups/condition is specified in the figure legends.

Behavioral, microdialysis/HPLC, and MRI/MSI experiments were performed as previously described^22–29^, and conducted at the end of 90-day treatment. Mice were then killed, and hippocampi were isolated for western blot (WB), cytokine assays, and RT-PCR analyses, whereas whole brains for immunohistochemistry were flash-frozen in 2-methylbutane. Biochemical analyses were performed as previously described^12^. The timeline of the experiments is described in Fig. 1a.

### Behavioral tests

The minimum interval between two consecutives procedures was 2 days. All tests were performed between 8:00 a.m. and 3:00 p.m., in a dimly lit condition. On the day of testing, the mice were acclimated for about 60 min in the behavioral room before the procedures were initiated. Mice were weighed every day during the entire period of the experiment. All behavioral tasks were analyzed by a blinded investigator.

#### Novel object recognition test (NORT)

Each mouse was habituated to an empty Plexiglas arena (45 × 25 × 20 cm) for 3 consecutive days. On training (day 4), mice were exposed to two identical objects (A+A) placed at opposite ends of the arena for 5 min. After 30 min and 24 h, the animals were subjected to a 5-min retention session where they were exposed to one object A and to a novel object B (after 30 min) and object C (after 24 h). Exploration was considered as pointing the head toward an object at a distance of <2.5 cm from the object, with its neck extended and vibrissae moving. Turning around, chewing, and sitting on the objects were not considered exploratory behaviors. Behavior was recorded with a MV750i camera (1024 × 768 resolution, Canon, Tokyo, Japan) and scored by a blinded investigator. Videotapes were analyzed as MPEG files using a behavioral tracking system furnished with infrared lighting-sensitive CCD cameras. Animal performances were monitored with the EthoVision XT version 7 video-tracking software system (Noldus Information Technology Inc., Leesburg, VA). The time of exploration was recorded, and an object recognition index (ORI) was calculated, such that ORI = (TN−TF)/(TN + TF), where TN and TF represent times of exploring the familiar and novel object, respectively. Mice that did not explore both objects during training were discarded from further analysis.

#### Inhibitory passive avoidance (IA)

On training, mice were placed in the fear-conditioning chamber and were allowed to explore for 2 min before receiving three electric foot shocks (1 s, 0.1 mA; intershock interval, 2 min). Animals were returned to the home cage 30 s after the last footshock. The animals were subsequently tested 24 h or 7 days after the training phase to assess the short- and long-term memory. During this phase, the behavior in the conditioning chamber was video recorded for 5 min and subsequently was analyzed for freezing behavior, which was defined as the absence of all movements except for respiration.

#### Morris water maze (MWM)

The test was conducted in a circular tank of 1.2 m in diameter, and locates in a room with several extra maze cues. Mice were trained to swim to a 14-cm-diameter circular Plexiglas platform submerged 1.5 cm beneath the surface of water and invisible to the mouse while swimming. The water temperature was kept at 25 °C throughout the duration of the test. The platform was fixed in place, equidistant from the center of the tank and its walls. Mice were subjected to four training trials per day and were alternated among four random starting points for 5 consecutive days. Mice were allowed to find and escape onto the submerged platform. If the mice failed to find the platform within 60 s, they were manually guided to the platform and were allowed to remain on it for 10 s. After this, each mouse was placed into a holding cage under a warming lamp for 25 s until the start of the next trial. Retention of the spatial memory (the probe trial) was assessed 1.5 and 24 h after the last training session and consisted of a 60 s trial without the platform. Mice were monitored by a camera mounted in the ceiling directly above the pool, and all trials were stored on videotape for subsequent analysis. The parameters measured during the probe trial included initial latency to cross the platform location, number of platform location crosses, and time spent in the target quadrant.

#### Tail suspension test (TST)

Mice were suspended for 6 min by the tail and the duration of immobility was measured during the last 4 min.

#### Forced swim test (FST)

Mice were individually placed in a Plexiglas cylinder (20 cm diameter, 50 cm high) containing 20 cm of water (25 °C). The experiment lasted for 6 min and the duration of immobility was analyzed during the last 4 min.

#### Sucrose preference test (SPT)

Singularly caged mouse had free access to two drinking bottles, the first filled with tap water, while the other with a 2% sucrose solution. Before the test, there was a period of adaptation that lasted for 48 h. The animals were then deprived of food and liquids for 3 h. During the next 24 h, free consumption of water and 2% sucrose solution took place, in the presence of *ad libitum* food. Fluid intake was measured afterward by weighing the drinking bottles. The sucrose preference (%) was determined as follows: sucrose solution intake (g)/total fluid intake (g) × 100.

### Biochemical testing procedures

#### RNA isolation and RT-PCR

Total RNA from hemi-hippocampi homogenates was extracted by using the NZY total RNA isolation kit (NZYTech, Lisboa, Portugal) following the company’s datasheet. The total RNA was measured by Nanodrop 1 000 spectrophotometer (Thermo Fisher Scientific, MD, USA), so, 1 µg of RNA was reverse transcribed to obtain cDNA by using oligo(dT) and random primers of the first-strand cDNA synthesis kit (NZYTech, Lisboa, Portugal). All PCRs were performed using supreme NZYTaq DNA polymerase (NZYTech, Lisboa, Portugal) with specific primers (Sigma-Aldrich, Milan, Italy) for tumor necrosis factor-α (TNF-α, forward primer 5′-CAGCCGATGGGTTGTACCTT-3′ and reverse primer 5′-CCGGACTCCGCAAAGTCTAA-3′), interleukin-1β (IL-1β, forward primer 5′-GGACCCCAAAAGATGAAGGGC-3′ and reverse primer 5′-GGAAAAGAAGGTGCTCATGTCC-3′), and IL-10 (forward primer 5′-GCCCTTTGCTATGGTGTCCT-3′ and reverse primer 5′-CTCTGAGCTGCTGCAGGAAT-3′). Glyceraldehyde 3-phosphate dehydrogenase (GAPDH, forward primer 5′-GCTACACTGAGGACCAGGTTGTC-3′ and reverse primer 5′-CCATGTAGGCCATGAGGTCCAC-3′) was used as reference gene.

#### Protein extraction and western blot analysis

Hemi-hippocampi were homogenized in ice-cold hypotonic lysis buffer (50 mM Tris/HCl, pH 7.5, 150 mM NaCl, 1 mM ethylenediaminetetraacetic acid (EDTA), 1% triton X-100, 1 mM phenylmethylsulfonyl fluoride (PMSF), 10 μg/ml aprotinin, and 0.1 mM leupeptin, all from Sigma-Aldrich, Milan, Italy) and incubated for 40 min at +4 °C. Protein dephosphorylation was avoided by adding a phosphatase inhibitor cocktail. The homogenates were then centrifuged, cellular membranes discarded, and the obtained supernatant was aliquoted and stored at −80 °C. Bradford assay was performed to calculate protein concentration. An equal amount of proteins (50 μg) was resolved on 12% acrylamide SDS-PAGE precast gels (Bio Rad Laboratories, Milan, Italy) and transferred onto nitrocellulose membranes through a semidry system (Bio Rad Laboratories, Milan, Italy). Membranes were blocked for 1 h either with no-fat dry milk or bovine serum albumin (BSA) powders in tris-buffered saline–0.1% tween 20 (TBS-T) (Tecnochimica, Rome, Italy).

Overnight incubation at +4 °C was performed with one of the following primary antibodies: rabbit anti-amyloid precursor protein (anti-APP 1:1 000, Cell Signaling, Danvers, MA, USA), rabbit anti-β-secretase (anti-BACE1 1:1 000, Cell Signaling, Danvers, MA, USA), mouse anti-β-amyloid (1:200, Millipore, Darmstad, Germany), rabbit anti-Akt (1:500, Cell Signaling, Danvers, MA, USA), rabbit anti-p[Thr308]Akt (1:5 000, Cell Signalling, Danvers, MA, USA), rabbit anti-glycogen synthase kinase-3β (anti-Gsκ-3β 1:1 000, Cell Signaling, Danvers, MA, USA), rabbit anti-p[Ser9]GSκ-3β (1:1 000, Cell Signaling, Danvers, MA, USA), rabbit anti-p[Ser396]tau (1:1 000, Thermo Fisher Scientific, Waltham, MA, USA), mouse anti-microtubule-associated protein 2 (anti-MAP2 1:250, Novus Biologicals, Littleton, CO, USA), rabbit anti-S100B (1:1 000, Epitomics, Burlingame, CA, USA), rabbit anti-glial fibrillary acidic protein (anti-GFAP 1:25 000, Abcam, Cambridge, UK), rabbit anti-p[Ser536]nuclear factor kappa-light-chain enhancer of activated B cells (anti-p[Ser536]NF-κB p65 1:2 000, Cell Signaling, Danvers, MA, USA), rabbit anti-inducible nitric oxide synthase (anti-iNOS, 1:8 000, Sigma-Aldrich, Milan, Italy), rabbit anti-glutamate transporter GLT-1 (1:1 000, Tocris, Bristol, UK), and mouse anti-glutamine synthetase clone GS-6 (1:1 000, Millipore, Darmstad, Germany). Rabbit anti-β-actin (1:1 500, Santa Cruz, Dallas, TX, USA) was used as loading control.

Membranes were incubated with a specific secondary horseradish peroxidase (HRP)-conjugated antibody (HRP-conjugated goat anti-rabbit IgG, 1:10 000–1:30 000; HRP-conjugated goat anti-mouse, 1:10 000; all from Jackson ImmunoResearch, Suffolk, UK) either in no-fat dry milk or BSA TBS-T. Immunocomplexes were detected by an enhanced chemiluminescence (ECL) kit (GE Healthcare Life Sciences, Milan, Italy) and the signal obtained was quantified by ImageJ software after densitometric scanning of the X-ray films (GE Healthcare Life Sciences, Milan, Italy).

#### Immunohistochemistry

Immunohistochemistry for GFAP and MAP2 was performed on coronal slices (12 µm thickness) containing the hippocampal regions, collected, and postfixed with 4% paraformaldehyde in 0.1 M phosphate buffer solution (PBS) (Tecnochimica, Rome, Italy). Slices were incubated with blocking solution and then incubated overnight in blocking solution containing either rabbit anti-GFAP (1:1 000, Abcam, Cambridge, UK) or mouse anti-MAP2 (1:250, Novus Biologicals, Littleton, CO, USA). Sections were incubated with the proper secondary antibody (1:200, fluorescein-affinipure goat anti-rabbit IgG (H+L); 1:300–1:400, rhodamine-affinipure goat anti-mouse IgG (H+L), Jackson ImmunoResearch, Suffolk, UK) and 4′,6-diamidino-2-phenylindole (DAPI 1:75 000, Sigma-Aldrich, Milan, Italy) in BSA at room temperature. Fluorescent signal was detected by an Eclipse E600 microscope (Nikon, Tokyo, Japan) using both Nikon Plan 10X/10.25 and Nikon Plan Fluor 20X/0.5 objectives. Pictures were captured by a QImaging camera (Canada) with NISelements BR 3.2 64-bit software with pixel resolution of 1024 × 1024.

Analysis was performed by ImageJ software and data were expressed as a ratio of the difference between the mean of fluorescence signal and the background (ΔF), and the non-immunoreactive regions (F_0_). To prevent any change in the fluorescent signal due to artifacts, the gain and time exposure were kept constant during all image acquisitions.

#### Cytokine array

Hippocampal homogenates were analyzed for cytokines presence using a mouse cytokine array panel A (R&D Systems, Minneapolis). A total of 100 μg for each hippocampal lysate were processed following the manufacturer’s instructions.

#### **In vivo** microdialysis and HPLC analysis

*In vivo* microdialysis was performed in awake and freely moving mice. Anesthetized mice were stereotaxically implanted with a CMA/7 guide cannula with stylet (CMA Microdialysis, Stockholm, Sweden) into the ventral hippocampus (anterior–posterior, −3.0 mm; lateral, +3.0 mm; and ventral, −1.8 mm from bregma). Following a 2-day recovery period, the CMA/7 probe was inserted and dialyses were carried out perfusing the probe with Krebs-Ringer phosphate (KRP) buffer at flow rate of 1 μl/min. The constituents of the KRP buffer were (in mM) NaCl 145, KCl 2.7, MgCl_2_ 1, CaCl_2_ 2.4, and Na_2_HPO_4_ 2, buffered at pH 7.4. After a 2-h stabilization period, four baseline samples were collected every 20 min. Probe position was verified histologically and glutamate was quantified by HPLC coupled to fluorescence detection.

#### Magnetic resonance imaging (MRI)/magnetic resonance spectroscopy (MRS)

Mice of 6 and 12 months undergo MRI and MRS scanning to evaluate genotype- and treatment-induced differences in brain metabolism. Animals were anesthetized with 2.5–1.5% isoflurane (IsoFlo, Abbott SpA, Berkshire, UK) in oxygen at flow rate of 1 l/min. MRI/MRS experiments were conducted on a 4.7T Agilent Inova preclinical system (Agilent Technologies Inc., Palo Alto, CA, USA) equipped with a combination of volume and surface coils (Rapid Biomedical GmbH, Rimpar, Germany). Fast spin-echo sagittal anatomical images (Repetition Time (TR)/Echo Time (TE) = 3200/60 ms, 13 slices of 0.8-mm thickness, Field of View (FOV) 20 × 25 mm^2^, matrix 256 × 256, 2 averages, and scan time 12 min) were acquired for positioning of the voxel for MRS. Single voxel localized 1H MR spectra (PRESS, TR/TE = 4 000/23 ms, NS = 256) were collected from hippocampus (volume 9.5 μl) according to a quantitative protocol, which includes water T2 measurements and LCModel fitting routine for spectral analysis^29^. We adopted the spectral analysis which considers Glx as the combined signal that mainly comes from glutamate plus glutamine because, at field strength generally used in human MRS studies, they have overlapping signals.

### Statistical analysis

Sample size was determined on the basis of our previous experiments and by using the software GPower. All data were expressed as mean ± standard error of measurement (SEM). Behavioral, biochemical, and MRI/MRS data were analyzed by two-way analyses of variance (ANOVA) with genotype (3×Tg-AD *vs* Non-Tg) and treatment (um-PEA *vs* placebo) as between-subject factors. Tukey’s honestly significant difference (HSD) test or Bonferroni’s test were used for multiple *post hoc* comparisons when required. The threshold for statistical significance was set at *p* < 0.05.

### Data availability

The data that support the findings of this study are available from the corresponding author upon reasonable request.

## Results

### Behavioral tasks

Statistical details are reported in Table 1.

**Table 1:** Results from the statistical analysis of data obtained from the behavioral tests of 6- and 12-month-old mice

Behavioral tests	Parameter	Genotype (G)	Treatment (T)	Interaction G × T
6 month old				
NORT	Object recognition index––30 min	*F* (1,38) = 0.377, n.s.	*F* (1,38) = 1.389, n.s.	*F* (1,38) = 6.406 *p* < 0.05
	Object recognition index––24 h	*F* (1,37) = 11.993, *p* < 0.01	*F* (1,37) = 2.617, n.s.	*F* (1,37) = 9.214, *p* < 0.01
IA	Latency to enter dark compartment—24 h	*F* (1,33) = 16.499, *p* < 0.001	*F* (1,33) = 5.513, *p* < 0.05	*F* (1,33) = 15.951, *p* < 0.001
	Latency to enter dark compartment—7 days	*F* (1,34) = 21.239, *p* < 0.001	*F* (1,34) = 29.431, *p* < 0.001	*F* (1,34) = 10.556, *p* < 0.01
MWM	Latency to cross platform location—1.5 h	*F* (1,39) = 6.842, *p* < 0.05	*F* (1,39) = 6.481, *p* < 0.05	*F* (1,39) = 8.757, *p* < 0.01
	Latency to cross platform location—24 h	*F* (1,39) = 4.698, *p* < 0.05	*F* (1,39) = 4.325, *p* < 0.05	*F* (1,39) = 7.879, *p* < 0.01
	Time in the target quadrant—1.5 h	*F* (1,39) = 4.441, *p* < 0.05	*F* (1,39) = 4.474, *p* < 0.05	*F* (1,39) = 6.050, *p* < 0.05
	Time in the target quadrant—24 h	*F* (1,39) = 4.836, *p* < 0.05	*F* (1,39) = 3.812, n.s.	*F* (1,39) = 4.994, *p* < 0.05
	Number of platf location crosses—1.5 h	*F* (1,39) = 4.835, *p* < 0.05	*F* (1,39) = 7.845, *p* < 0.01	*F* (1,39) = 5.634, *p* < 0.05
	Number of platf location crosses—24 h	*F* (1,39) = 3.454, n.s.	*F* (1,39) = 3.660, n.s.	*F* (1,39) = 4.633, *p* < 0.05
TST	Immobility	*F* (1,39) = 44.062, *p* < 0.001	*F* (1, 39) = 7.203, *p* < 0.05	*F* (1, 39) = 9.308, p < 0.01
FST	Immobility	*F* (1,39) = 28.558, p < 0.001	*F* (1, 39) = 5.473, p < 0.05	*F* (1, 39) = 11.442, *p* < 0.01
SPT	Total fluid intake	*F* (1,39) = 0.349, n.s.	*F* (1,39) = 0.382, n.s.	*F* (1,39) = 1.991, n.s.
	Sucrose preference	*F* (1,39) = 22.547, *p* < 0.001	*F* (1,39) = 3.608, n.s.	*F* (1,39) = 8.933, *p* < 0.01
12 month old				
NORT	Object recognition index—30 min	*F* (1,30) = 0.154, n.s.	*F* (1,30) = 1.251, n.s.	*F* (1,30) = 9.460, *p* < 0.01
	Object recognition index—24 h	*F* (1,31) = 9.310, *p* < 0.01	*F* (1,31) = 0.112, n.s.	*F* (1,31) = 3.431, n.s.
IA	Latency to enter dark compartment—24 h	*F* (1,29) = 4.799, *p* < 0.05	*F* (1,29) = 0.261, n.s.	*F* (1,29) = 2189, n.s.
	Latency to enter dark compartment—7 days	*F* (1,29) = 1653, n.s.	*F* (1,29) = 0179, n.s.	*F* (1,29) = 1723, n.s.
MWM	Latency to cross platform location—1.5 h	*F* (1,29) = 1273, n.s.	*F* (1,29) = 3.760, n.s.	*F* (1,29) = 4.326, *p* < 0.05
	Latency to cross platform location—24 h	*F* (1,29) = 5.725, *p* < 0.05	*F* (1,29) = 0.388, n.s.	*F* (1,29) = 0.464, n.s.
	Time in the target quadrant—1.5 h	*F* (1,29) = 3.715, *p* = 0.065	*F* (1,29) = 3.022, *p* = 0.094	*F* (1,29) = 3.437, *p* = 0.07
	Time in the target quadrant—24 h	*F* (1,29) = 2.107,n.s	*F* (1,29) = 1.303, n.s.	*F* (1,29) = 1.277, n.s.
	Number of platf location crosses—1.5 h	*F* (1,29) = 1.783, n.s.	*F* (1,29) = 1.030, n.s.	*F* (1,29) = 6.303, *p* < 0.05
	Number of platf location crosses—24 h	*F* (1,29) = 4.474, *p* < 0.05	*F* (1,29) = 0.261, n.s.	*F* (1,29) = 0.225. n.s.
TST	Immobility	*F* (1,28) = 11.873, *p* < 0.01	*F* (1,28) = 0.363, n.s.	*F* (1,28) = 1.681, n.s.
FST	Immobility	*F* (1,29) = 39.686, *p* < 0.001	*F* (1,29) = 0.00321, n.s.	*F* (1, 29) = 4.690, *p* < 0.05
SPT	Total fluid intake	*F* (1,29) = 2.638, n.s.	*F* (1,29) = 0.0558, n.s.	*F* (1,29) = 0.733, n.s.
	Sucrose preference	*F* (1,29) = 1.126, n.s.	*F* (1,29) = 2.319, n.s.	*F* (1,29) = 4.563, *p* < 0.05

Two-way analyses of variance (ANOVA) with genotype (3×Tg-AD *vs* Non-Tg) and treatment (um-PEA *vs* placebo) as between-subject factors (*n* = 10–12 per group). Details are reported in the text

*NORT* novel object recognition test, *IA* inhibitory passive avoidance, *MWM* Morris water maze, *TST* tail suspension test, *FST* forced swim test, *SPT* sucrose preference test

#### Um-PEA improves learning and memory in 6-month-old 3×Tg-AD mice

We tested the effects of um-PEA on both short- (30 min) and long-term (24 h) memory with a novel object recognition test (NORT). Two-way ANOVA analysis revealed significant changes in the time mice spent exploring the new object across the four different groups. At 30 min, we found significant genotype-by-treatment interaction effects, while no significant differences were found for the main effects of genotype and treatment. *Post hoc* comparisons showed a significant higher object recognition index (ORI) for 3×Tg-AD mice treated with um-PEA with respect to placebo-treated 3×Tg-AD mice (Fig. 1b). Performing this trial 24 h later, we observed a significant genotype and genotype-by-treatment interaction effect of ORI in the exploration session among the four groups. *Post hoc* analysis demonstrated that um-PEA-treated 3×Tg-AD mice performed significantly better than the placebo-treated 3×Tg-AD group, and indicated that, at both time points, um-PEA had no effect on the performance of Non-Tg mice (Fig. 1b).

Contextual learning and memory were then evaluated by an inhibitory passive avoidance task (IA), and the retention test was then conducted 24 h or 7 days after the training trial to assess short- and long-term memory. Statistical analysis indicated, at both time points, a significant main effect of genotype, treatment, and genotype-by-treatment interaction. Multiple *post hoc* comparisons indicated that um-PEA-treated transgenic mice performed better than placebo-treated 3×Tg-AD mice, and reached the same performance level of Non-Tg animals (Fig. 1c). This was not due to differences in or enhanced sensitivity to the footshock, as all mice had similar jump responses upon shock administration.

Spatial learning was measured by the Morris water maze (MWM). Mice received four training trials/day for 5 consecutive days to locate the hidden platform. Statistics demonstrated no difference in spatial memory during 5 days of training among all groups measured (data not shown). To determine the effects of um-PEA on memory, the platform was removed from the maze, and tests were conducted 1.5 or 24 h following the last training trial to independently assess both short- and long-term memory, respectively. Um-PEA rescued the early spatial memory deficits present in 6-month-old 3×Tg-AD mice, as indicated by a significantly decreased latency to cross the platform location of the 3×Tg-AD mice treated with um-PEA compared to placebo-treated 3×Tg-AD (Fig. 1d). Moreover, the number of platform location crosses, as well as the time spent in the target quadrant were significantly increased in um-PEA-treated 3×Tg-AD compared to placebo-treated 3×Tg-AD mice. Multiple *post hoc* comparisons showed that 3×Tg-AD mice treated with um-PEA performed similarly to the Non-Tg mice in all probe trials and at both time points. Finally, um-PEA had no significant effects on learning or memory retention in Non-Tg mice (Fig. 1e, f).

Overall, these data indicate that um-PEA treatment rescues early learning and memory deficits in 6-month-old 3×Tg-AD mice.

#### Um-PEA improves short-term learning and memory alone in 12-month-old 3×Tg-AD mice

Two-way ANOVA for the ORI at a time point of 30 min (short memory) in 12-month-old mice revealed a significant genotype-by-treatment interaction effect, while no significant main effects of genotype and treatment were found. Multiple *post hoc* comparisons showed a significant higher ORI in um-PEA-treated 3×Tg-AD with respect to placebo-treated 3×Tg-AD mice (Fig. 1g).

When the probe trial was performed 24 h after the exploration session (long-term memory), we observed a significant main effect of genotype only among the four groups (Fig. 1g).

In 12-month-old mice, IA showed significant main effects only for genotype 24 h after the training trial, whereas at 7 days, no significant main effects were observed (Fig. 1h).

The results from MWM in 12-month-old mice demonstrated that no significant differences were observed during 5 days of training among the four different groups (data not shown). When the probe trial was performed 1.5 h after the last training session (short-term memory), statistical analysis showed that um-PEA significantly decreased the latency to cross the platform location and increased the number of platform location crosses in 3×Tg-AD compared to placebo-treated 3×Tg-AD mice (Fig. 1i, j). Regarding the time spent in the target quadrant at 1.5 h after the last training session, no significant differences were observed among the four groups, although um-PEA induced a trend toward an increase (+43%) in the 3×Tg-AD compared to placebo-treated 3×Tg-AD mice (Fig. 1k). Moreover, when testing was performed at 24 h after the last training session (long-term memory), statistical analysis showed a significant main effect of genotype alone for both latency to cross the platform location and the number of platform location crosses (Fig. 1i–k).

Overall, these data indicate that um-PEA improves the short-term memory of 12-month-old 3×Tg-AD mice, with no significant effects on long-term memory. Moreover, um-PEA exerts no significant effects on learning or memory in aged Non-Tg mice.

#### Um-PEA ameliorates the depressive- and anhedonia-like behaviors in 3×Tg-AD mice

Depressive-like behaviors were measured by the tail suspension test (TST) and forced swim test (FST). At 6 months of age, significant main effects of treatment, genotype, and genotype-by-treatment interactions were observed. *Post hoc* comparisons revealed that the immobility time in both tests was higher in placebo-treated 3×Tg-AD than in placebo-treated Non-Tg mice. Moreover, um-PEA significantly decreased the immobility time in the 3×Tg-AD for both tests (Fig. 1l, m). Interestingly, we found a significant main effect of genotype alone in 12-month-old mice, with no significant main effects of treatment and genotype-by-treatment interaction (Fig. 1l, m).

Anhedonia-like behaviors were measured by a sucrose preference test (SPT). SPT revealed a significant main effect of genotype and genotype-by-treatment interaction at 6 months of age, and other significant main effects included a genotype-by-treatment interaction at 12 months of age (Fig. 1n, o). Multiple *post hoc* comparisons demonstrated a significantly increased preference for sucrose in the placebo-treated Non-Tg mice compared to placebo-treated 3×Tg-AD mice. Interestingly, um-PEA restored the preference for the sweet solution in the 3×Tg-AD group at both 6 and 12 months of age. This effect was not accounted for by a difference in total fluid intake among all groups (Fig. 1n, o).

Altogether, these results suggest that 3×Tg-AD mice show a depressive-like phenotype that is reversed by um-PEA treatment only at 6 months of age, while no significant effect is observed at 12 months of age. Differently, um-PEA treatment attenuates the anhedonia-like phenotype of both 6- and 12-month-old 3×Tg-AD mice. Moreover, um-PEA has no significant effect on Non-Tg mice.

#### Um-PEA reduces Aβ formation in aged 3×Tg-AD mice

**Fig. 2:**
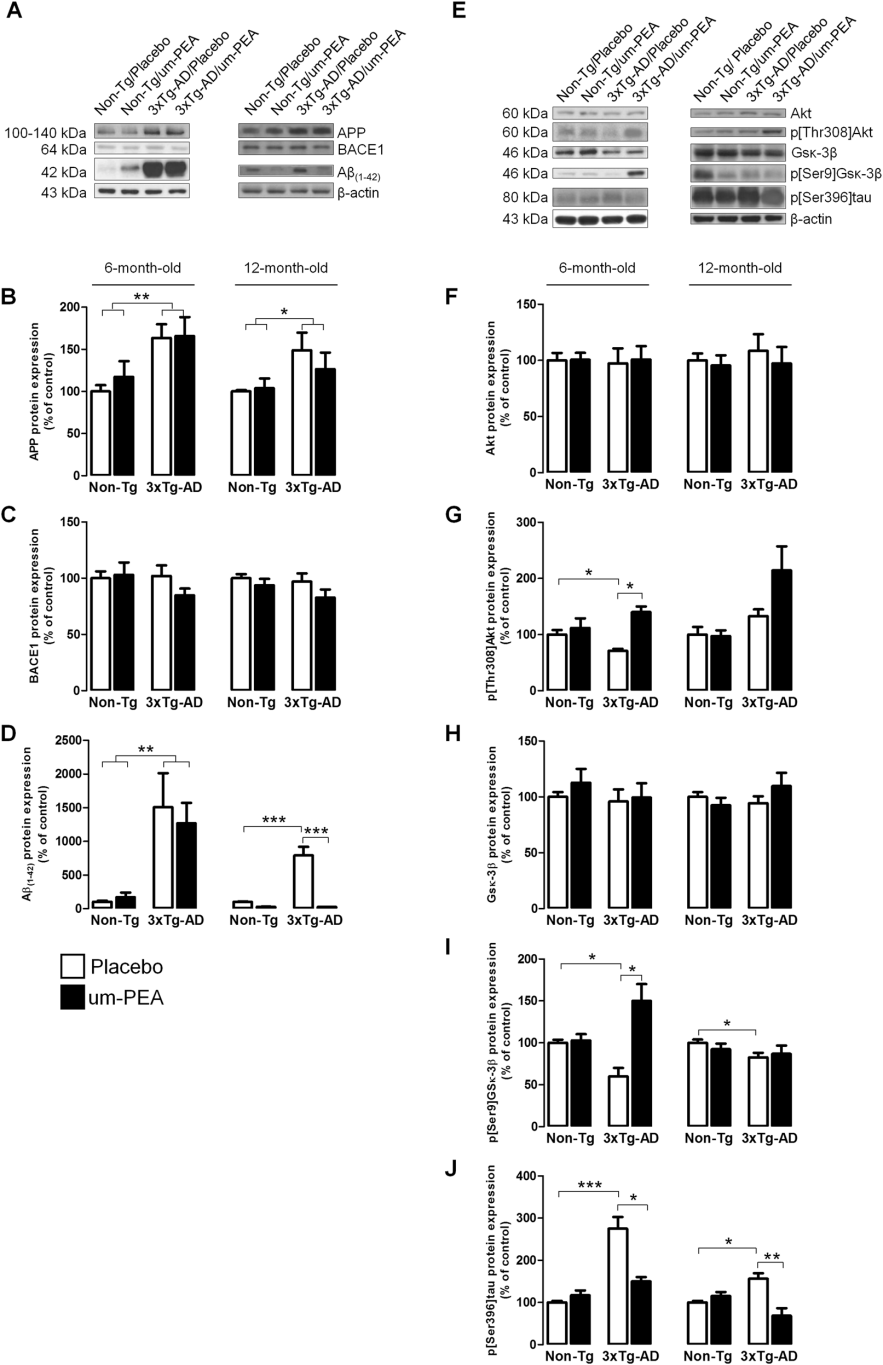
Um-PEA effects on AD pathology. Evaluation of protein expression in hippocampi of 6- and 12-month-old 3×Tg-AD and age-matched Non-Tg mice chronically treated with placebo (open bars) or um-PEA (black bars). (**a**) Representative western blots for APP, BACE1, and Aβ_(1–42)_ proteins and (**b–d**) densitometric analyses normalized to β-actin used as loading controls (*N* = 3, in triplicate). (**e**) Representative western blots for Akt, p[Thr308]Akt, Gsκ-3β, p[Ser9]Gsκ-3β, p[Ser396]tau, and (**f–j**) densitometric analysis normalized to β-actin used as loading control (*N* = 3, in triplicate). The results are expressed as percentage of control (Non-Tg/placebo groups). The data are presented as means ± SEM. Statistical analysis was performed by two-way ANOVA followed by Bonferroni’s multiple-comparison test (*p < 0.05; **p < 0.01; ***p < 0.001)

To evaluate the effect of um-PEA on AD-like pathology, we studied the expression of APP, BACE1, and Aβ_(1–42)_. Placebo-treated 3×Tg-AD mice of 6 and 12 months compared with placebo-treated Non-Tg littermates showed a significant increase of APP and, despite no changes in BACE1 expression, exhibited a massive increase in Aβ_(1–42)_ levels. At both ages, um-PEA did not change the expression of full-length APP. Interestingly, um-PEA strongly reduced Aβ_(1–42)_ expression in 3×Tg-AD mice only at 12 months of age (Fig. 2a–d).

Together, these results show that chronic um-PEA treatment reduces hippocampal Aβ_(1–42)_ expression in aging 12-month-old 3×Tg-AD mice, while inducing no significant effects in younger 6-month-old 3×Tg-AD mice.

#### Um-PEA reduces tau phosphorylation and promotes neuronal survival in 3×Tg-AD mice

The expression of Gsκ-3β and the downstream abnormally phosphorylated (p[Ser396]tau) tau protein, both closely related to NFT formation and its associated neuronal impairments, were evaluated^30–34^. We also assessed the expression of the Akt, a kinase whose active form p[Thr308]Akt is responsible for Gsκ-3β inactivation through Ser9 phosphorylation^35^. The results showed, despite no changes in the total Akt quantity, a significant decrease of p[Thr308]Akt in 6-month-old placebo-treated 3×Tg-AD mice in comparison with placebo-treated Non-Tg littermates. Although no differences in Gsκ-3β total amount were detected, placebo-treated 3×Tg-AD demonstrated a significant p[Ser9]Gsκ-3β reduction at both ages compared to placebo-treated Non-Tg mice. Um-PEA increased both p[Thr308]Akt and p[Ser9]Gsκ-3β in 6-month-old 3×Tg-AD compared to age-matched Non-Tg mice. Moreover, in placebo-treated 3×Tg-AD mice at both ages, we observed a significant p[Ser396]tau increase that was substantively reduced by um-PEA (Fig. 2e–j).

**Fig. 3:**
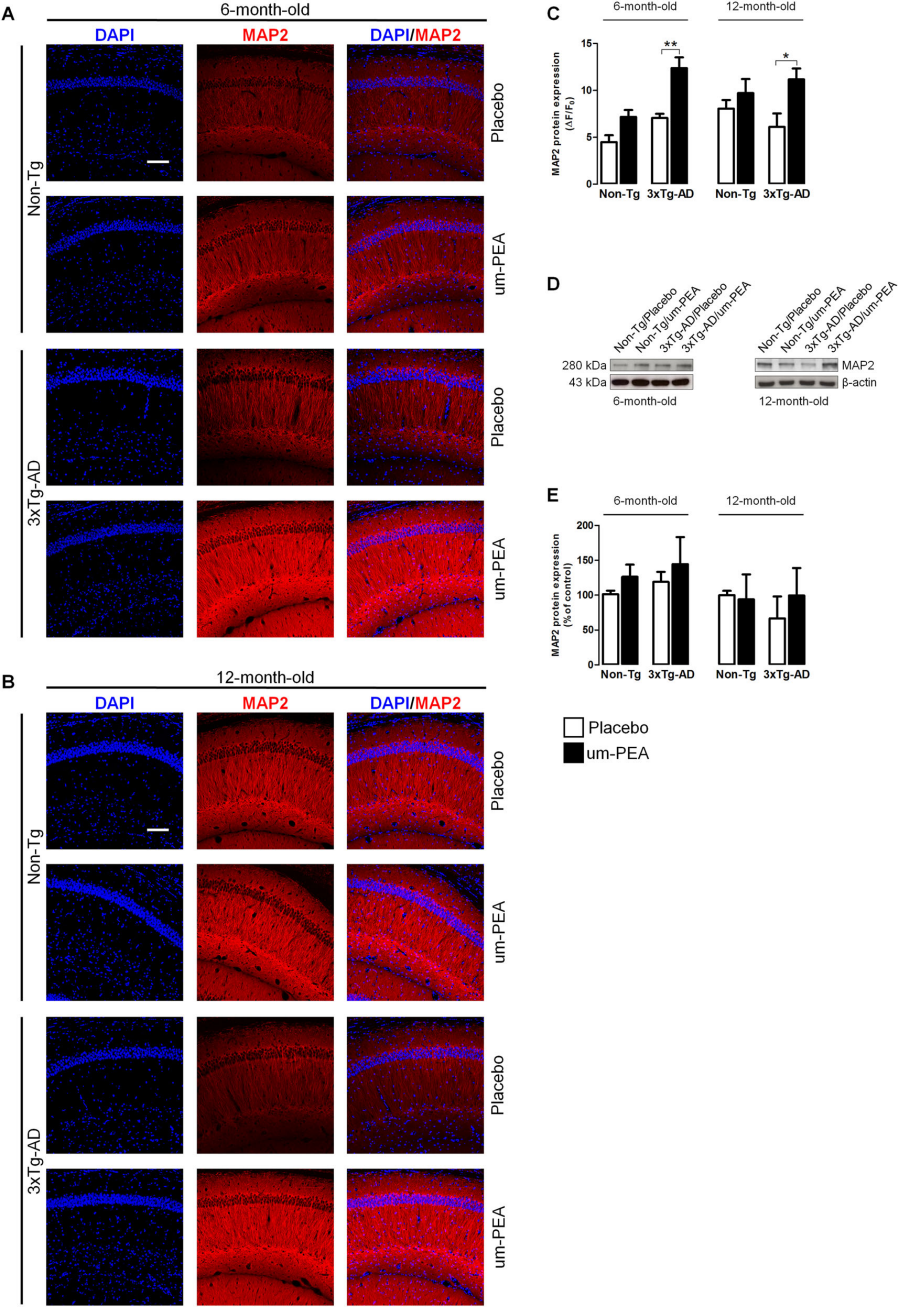
Um-PEA effects on neuronal viability in hippocampus of 3×Tg-AD and Non-Tg mice. Evaluation of neuronal marker expression in hippocampi of 6- and 12-month-old 3×Tg-AD and age-matched Non-Tg mice chronically treated with placebo (open bars) or um-PEA (black bars). (**a**, **b**) Representative fluorescent photomicrographs of microtubule-associated protein 2 (MAP2) (red) staining in the CA1 region of hippocampi at both 6 and 12 months of age, and (**c**) fluorescence analysis expressed as ΔF/F_0_. Nuclei were stained with DAPI (blue) (*N* = 3, in triplicate). (**d**) Representative western blots for MAP2 and (**e**) densitometric analyses normalized with β-actin used as loading controls (*N* = 3, in triplicate). The results are expressed as percentage of control (Non-Tg/placebo groups). The data are presented as means ± SEM. Statistical analysis was performed by two-way ANOVA followed by Bonferroni’s multiple-comparison test (*p < 0.05; p** < 0.01). Scale bar 100 µm

As a consequence of these observations, we investigated neuronal survival by testing the expression of MAP2. Western blot experiments did not show any difference between expression in any of these groups at both ages (Fig. 3d, e). However, immunofluorescence experiments, which were performed to examine subtle differences in expression between different hippocampal subregions, revealed a significant increase in MAP2 immunoreactivity in the CA1 of um-PEA-treated 3×Tg-AD mice at both ages (Fig. 3a–c).

Altogether, these data suggest that um-PEA reduces abnormal tau phosphorylation in the hippocampus of 3×Tg-AD mice at 6 and 12 months of age, and that such an effect may be partially mediated by the Gsκ-3β/Akt pathway. Moreover, um-PEA promotes MAP2 expression and hence neuronal survival in the CA1 subregion of the hippocampus of 3×Tg-AD mice.

#### Um-PEA normalizes astrocyte function and restrains neuroinflammation

**Fig. 4:**
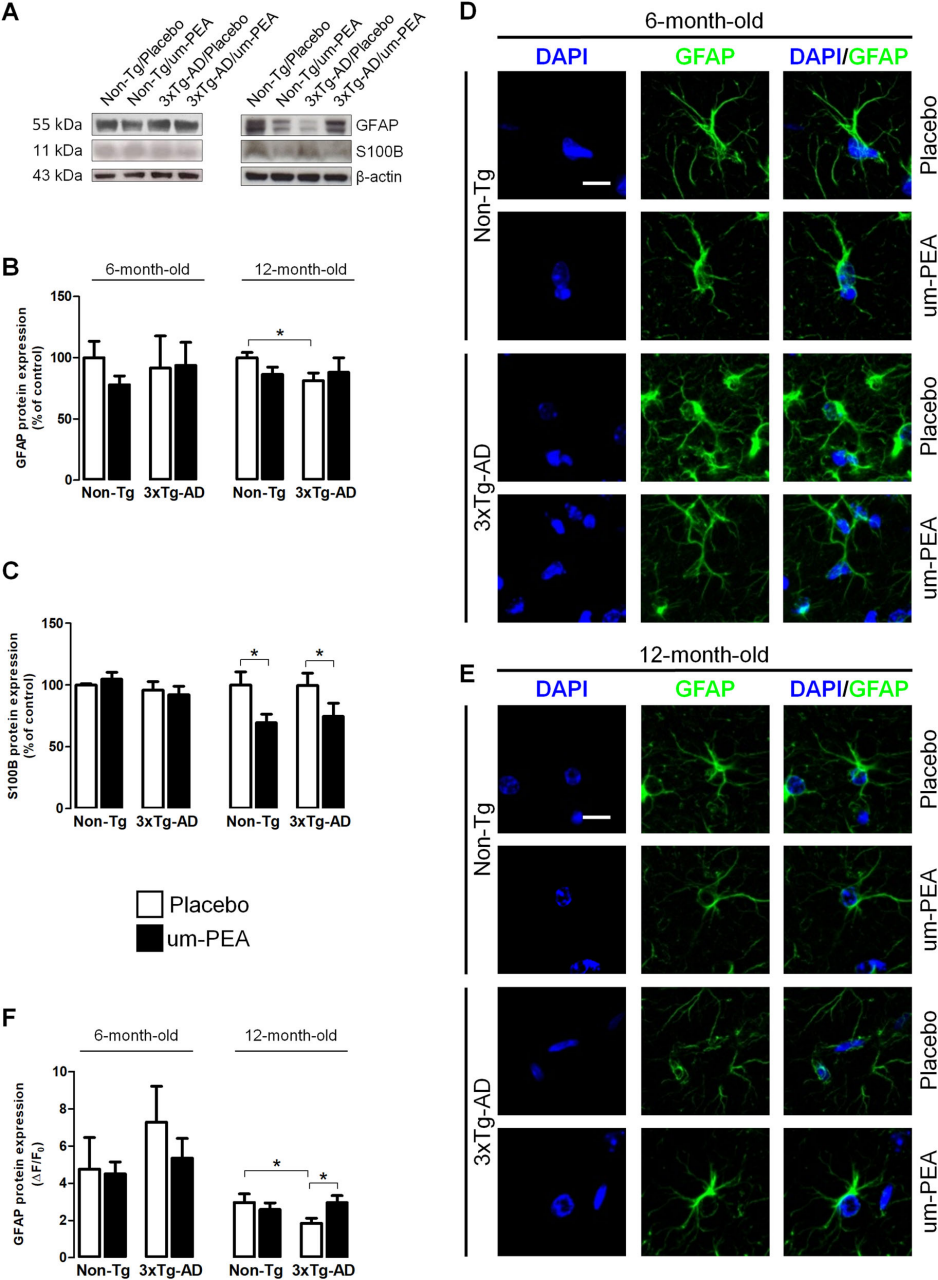
Um-PEA effects on astrocyte functionality in hippocampus of 3×Tg-AD and non-Tg mice. Evaluation of astrocytic markers in hippocampi of 6- and 12-month-old 3×Tg-AD and age-matched Non-Tg mice chronically treated with placebo (open bars) or um-PEA (black bars). (**a**) Representative western blots for GFAP and S100B proteins and (**b**, **c**) densitometric analyses normalized with β-actin used as loading controls. Results are expressed as percentage of control (Non-Tg/placebo groups) (*N* = 3, in triplicate). (**d**, **e**) Representative fluorescent photomicrographs of GFAP (green) staining in the CA1 subregion of hippocampi at both 6 and 12 months of age, and (**f**) fluorescence analysis expressed as ΔF/F_0_. Nuclei were stained with DAPI (blue) (*N* = 3, in triplicate). The data are presented as means ± SEM. Statistical analysis was performed by two-way ANOVA followed by Bonferroni’s multiple-comparison test (*p < 0.05). Scale bar 10 µm

We examined the effects of chronic um-PEA treatment on astrocyte function. Specifically, the expression of the cytoskeletal GFAP and S100B, a glial-derived neurotrophin whose levels are affected in AD^6^, was tested. We did not detect any significant difference in 6-month-old 3×Tg-AD mice compared to Non-Tg littermates in the expression of both GFAP and S100B (Fig. 4a–d, f).

**Fig. 5:**
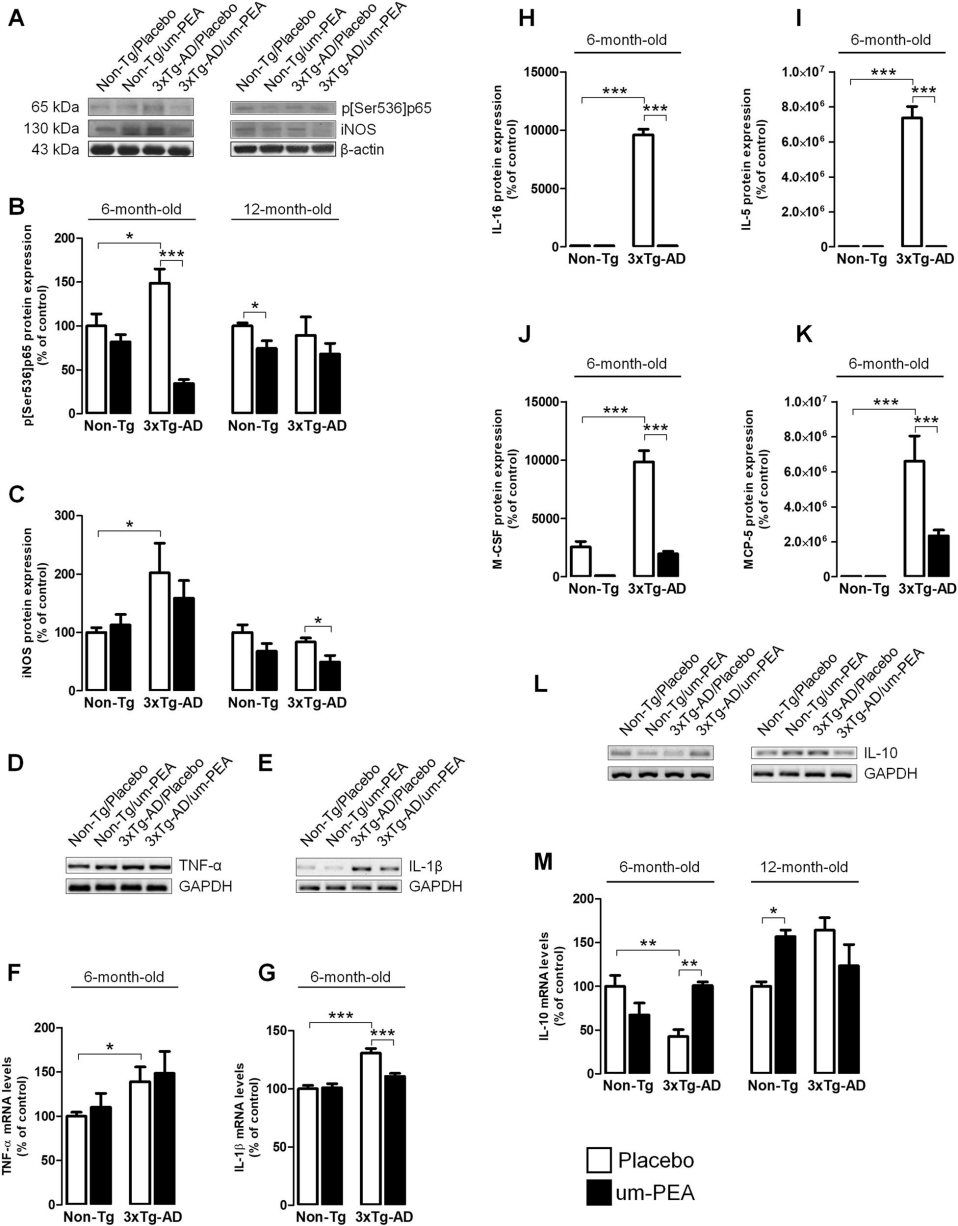
Um-PEA effects on neuroinflammation in hippocampus of 3×Tg-AD and Non-Tg mice. Evaluation of proinflammatory markers in hippocampi of 6- and 12-month-old 3×Tg-AD and age-matched Non-Tg mice chronically treated with placebo (open bars) or um-PEA (black bars). (**a**) Representative western blots for p[Ser536]p65 and iNOS proteins and (**b**, **c**) densitometric analyses normalized with β-actin used as loading controls. Results are expressed as percentage of control (Non-Tg/placebo groups) (*N* = 3, in triplicate). (**d**, **e**) Representative results obtained from RT-PCR in 6-month-old mice for tumor necrosis factor-α (TNF-α) and interleukin-1β (IL-1β), and (**f**, **g**) densitometric analysis of corresponding bands normalized with glyceraldehyde 3-phosphate dehydrogenase (GAPDH). Results are expressed as percentage of control (Non-Tg/placebo groups) (*N* = 3, in triplicate). (**h–k**) Densitometric analysis of cytokine array for IL-16, IL-5, macrophage colony-stimulating factor (M-CSF), and monocyte chemotactic protein 5 (MCP-5). Results are expressed as percentage of control (Non-Tg/placebo groups) (*N* = 3, in triplicate). (**l**) Representative results obtained from RT-PCR in 6- and 12-month-old mice for IL-10 and (**m**) densitometric analysis of corresponding bands normalized with glyceraldehyde 3-phosphate dehydrogenase (GAPDH). Results are expressed as percentage of control (Non-Tg/placebo groups) (*N* = 3, in triplicate). The data are presented as mean ± SEM. Statistical analysis was performed by two-way ANOVA followed by Bonferroni’s multiple-comparison test (*p < 0.05; **p < 0.01; ***p < 0.001)

We then examined the expression of a range of cytokines which revealed that 3×Tg-AD mice exhibit a transition to an increased proinflammatory state. It is notable that placebo-treated 6-month-old 3×Tg-AD mice displayed an increased transactivation of NF-κB p65 subunit, an overproduction of proinflammatory mediators, including iNOS, TNF-α, IL-1β, IL-16, and IL-5, macrophage colony-stimulating factor (M-CSF), monocyte chemotactic protein 5 (MCP-5), and reduction of the anti-inflammatory IL-10. Chronic treatment with um-PEA almost completely abolished the increase in inflammatory markers observed in 6-month-old 3×Tg-AD mice, and suppressed the expression of p[Ser536]p65, IL-1β, M-CSF, IL-16, MCP-5, and IL-5 but not iNOS and TNF-α, while enhancing IL-10 transcription (Fig. 5).

Contrasting results were obtained in aging animals. Indeed, placebo-treated 12-month-old 3×Tg-AD mice exhibited a significant reduction of GFAP expression compared to placebo-treated Non-Tg (Fig. 4a, b), and immunofluorescence revealed that um-PEA restored GFAP expression in the CA1 subregion of the hippocampus (Fig. 4e, f). Moreover, we did not observe any genotype-related difference in S100B expression. Interestingly, um-PEA significantly decreased S100B levels in both genotypes (Fig. 4a, c). Finally, in 12-month-old mice, we did not detect a transition to a proinflammatory state. However, at this age, um-PEA reduced iNOS expression in 3×Tg-AD, and enhanced IL-10 transcript in Non-Tg (Fig. 5a, c, l, m).

Collectively, these results show that 6-month-old 3×Tg-AD mice exhibit mild astrocyte activation but displayed an intense inflammatory status. In contrast, 12-month-old 3×Tg-AD mice did not demonstrate astrocyte activation but a slight astrocyte atrophy not accompanied by neuroinflammation. Um-PEA stabilizes the altered parameters related to astrocyte function, bringing them to more physiological levels, and restrains neuroinflammation.

#### Um-PEA effect on glutamatergic transmission

**Fig. 6:**
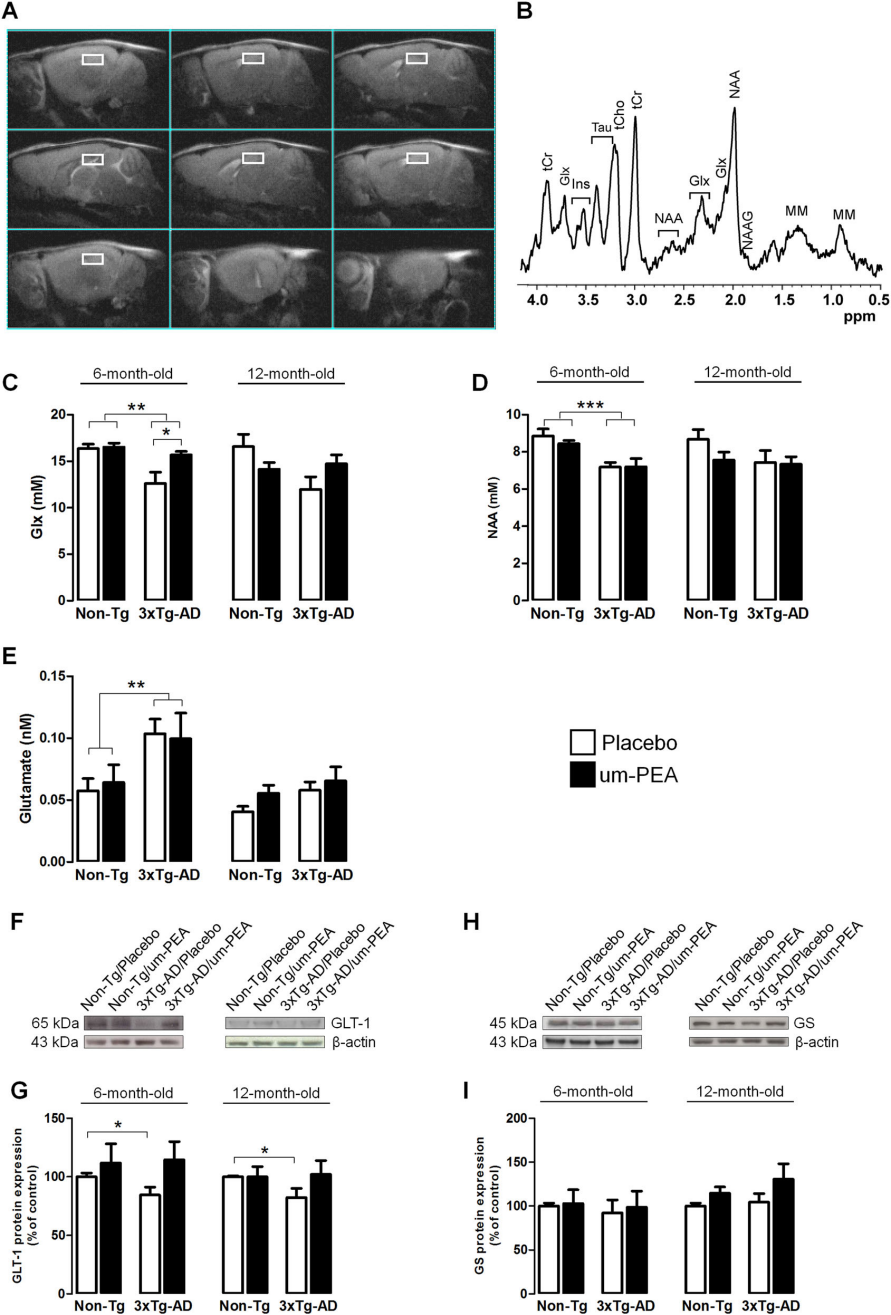
Um-PEA effects on brain metabolism and the glutamatergic system. Evaluation of the glutamatergic system in hippocampi of 6- and 12-month-old 3×Tg-AD and age-matched Non-Tg mice chronically treated with placebo (open bars) or um-PEA (black bars). (**a**) MRI panel—Example of *in vivo* fast spin-echo sagittal anatomical images (Repetition Time (TR)/Echo Time (TE) = 3200/60 ms, consecutive slices). Voxels localized on hippocampus are indicated by the white rectangles. (**b**) MRS panel—Examples of in vivo 1 H spectra (PRESS, TR/TE = 4000/23 ms, NS = 256). Metabolite assignments: inositol (Ins), total creatine (tCr), glutamine + glutamate (Glx), taurine (Tau), total choline (tCho), N-acetyl-aspartate (NAA), N-acetyl-aspartyl-glutamate (NAAG), and macromolecules (MM). Histograms showing (**c**) Glx and (**d**) NAA hippocampal concentrations at 6 and 12 months of age, respectively, expressed in nM (*N* = 12). (**e**) Results from HPLC on extracellular glutamate hippocampal concentrations at 6 and 12 months of age of Non-Tg and 3×Tg-AD mice. (**f**, **h**) Representative western blots for glutamate transporter 1 (GLT-1) and glutamine synthetase (GS) proteins at 6 and 12 months of age, and (**g**, **i**) densitometric analyses normalized with β-actin used as loading controls (*N* = 3, in triplicate). Results are expressed as percentage of control (Non-Tg/placebo groups). Data are presented as means ± SEM. Statistical analysis was performed by two-way ANOVA followed by Bonferroni’s multiple-comparison test (*p < 0.05; **p < 0.01; ***p < 0.001)

Quantitative magnetic resonance spectroscopy (MRS) analyses demonstrated that 6-month-old 3×Tg-AD mice had reduced hippocampal levels of both Glx (a combined measure of glutamate and glutamine) and N-acetyl-aspartate (NAA) compared to age-matched Non-Tg littermates. Interestingly, um-PEA significantly increased Glx levels only in the 3×Tg-AD mice (Fig. 6a–d). As Glx concentration reflects both intracellular and extracellular glutamate and glutamine pools, we attempted to gain deeper insights into the functional state of glutamatergic transmission by microdialysis. The results showed that basal extracellular glutamate levels in the hippocampus of 6-month-old 3×Tg-AD were significantly higher compared to age-matched Non-Tg mice (Fig. 6e). Westren blot analyses indicated that the higher extracellular glutamate levels in transgenic mice may be partially due to the reduced expression of GLT-1, while the expression of GS remained unaffected (Fig. 6f–i). Even though our results were not statistically significant, um-PEA treatment induced a trend toward an increase of GLT-1 (+35%) in 6-month-old 3×Tg-AD mice compared to placebo-treated Non-Tg ones (Fig. 6g, h).

In 12-month-old mice, we did not record any genotype-related differences in Glx, NAA, or extracellular glutamate levels despite a reduction in GLT-1 being evident (Fig. 6).

Collectively, these results indicate that 6-month-old 3×Tg-AD mice exhibit a disruption to glutamatergic function and that um-PEA increases Glx levels.

## Discussion

This study, employing a multidisciplinary approach, provides compelling evidence that a chronic um-PEA treatment exerts anti-inflammatory and neuroprotective effects in a murine model of AD that recapitulates the salient neural and cognitive impairments seen in this disease. It also demonstrates that um-PEA exerts these effects in both mildly declining young (6-month-old) and severely declining aging (12-month-old) 3×Tg-AD mice, which suggests its potential to arrest the decline in neural and cognitive function at two separate stages of the disease. These data also provide novel insights into the molecular mechanisms involved in AD. We comprehensively examined a number of pathways underlying astrocyte function, neuroinflammation, and neuronal integrity in 6- and 12-month-old 3×Tg-AD mice chronically receiving um-PEA or placebo for 3 months. Our results confirm that important behavioral and molecular modifications occur during the early stages of AD, and demonstrate that chronically administered um-PEA restrains or reverses most of them.

These results are important as AD has important public health and economic consequences and there is a need to develop novel effective therapeutic strategies to complement or replace those in current use. It has been apparent for a long time that PEA displays marked anti-inflammatory properties in peripheral inflammation models and it has demonstrated high effectiveness in a number of neurodegenerative disorders that present with an inflammatory component, including AD, Parkinson’s disease, and multiple sclerosis^10,14,36,37^. The recent availability of um-PEA, a crystalline form on micrometric size which improves both its pharmacokinetics and bioavailability^19,38^, prompted us to test its anti-inflammatory and neuroprotective effects in 3×Tg-AD mice.

First, we found that um-PEA rescued the early hippocampal, cortex, and amygdala-dependent memory impairments seen in 3×Tg-AD mice. The youngest mice (6-month old) demonstrated the largest significant improvement of both short- and long-term memory. Less clear-cut effects were seen in the oldest mice (12-month old), where um-PEA improved short-term memory (or at least some behavioral parameters, e.g., ORI, and latency and number of crosses in the MWM), but did not induce significant effects on long-term memory. This study also demonstrates that um-PEA induces other noncognitive effects that are relevant to AD. In this regard, following the observation that a depressive-like phenotype is present in the same strain of 18-month-old 3×Tg-AD mice^24^, we identified that this phenotype is already present by 6 months of age and is reversible at 6 but not at 12 months of age. However, we found that um-PEA attenuates a similar anhedonia-like phenotype of both young (6-month-old) and aging (12-month-old) 3×Tg-AD mice.

It is widely accepted that glia mediate the response to several brain injuries, including the deposition of Aβ, and undergo important morphological and functional changes that can, in turn, influence the progression of the disease. These modifications are intricate and heterogeneous, and can be crudely classified into hyperreactivity or atrophy^39,40^. Moreover, a direct correlation has been established between glial dysfunction and the induction of proinflammatory pathways^41^. In younger mice, although we did not detect significant alterations of GFAP and S100B, the best-known markers of astrocytic activation, we found that a range of parameters suggesting an increase in neuroinflammation occurred. These were reduced by um-PEA, suggesting that it restrains the transition to a neuroinflammatory environment. A comparison of results suggests that um-PEA effects are completely different in 3×Tg-AD mice at 6 and 12 months of age. At 12 months, mice do not show any noteworthy signs of neuroinflammation and merely displayed moderate astrocyte atrophy.

Based on our results and the hypothesis that neuroinflammation accelerates the development of the AD phenotype, increasing Aβ accumulation, we explored the main elements involved in the pro-amyloidogenic pathway^42^. 3×Tg-AD mice express a mutant APP_swe_ transgene, and we recorded an accumulation of this protein in these mice at both 6 and 12 months. No accumulation of BACE1 protein occurred, however, a 15-fold higher increase in Aβ_(1–42)_ expression was observed in the 3×Tg-AD compared to Non-Tg mice. Our results demonstrated the ability of um-PEA to strongly suppress Aβ_(1–42)_ expression in 12-month-old 3×Tg-AD mice, suggesting an important pathway by which it can restrain the disease progression. Another iconic hallmark of AD is the formation of NFTs which are mainly caused by the abnormal phosphorylation of tau. Tau protein is a microtubule-associated protein that facilitates microtubule assembly and stabilization as a function of its phosphorylation state^30^. However, under pathological conditions, including AD, enhanced phosphorylation (known as “hyperphosphorylation”) occurs, which leads to microtubule dissociation, aggregation into paired helical filaments, and the subsequent production of insoluble NFTs^43,44^. These events worsen axonal transport and synaptic function, which facilitates the development of a neurotoxic environment and cognitive impairments^31,32^. Several kinases add phosphate groups to tau-specific amino acids and Gsκ-3β is one of the primary kinases that are involved^30^ and its activity is regulated by Akt, a serine/threonine-specific kinase^34^. We observed significant alterations of the Akt/Gsκ-3β pathway in 3×Tg-AD mice, at both mild (6-month-old) and severe (12-month-old) stages of pathology, and have also demonstrated for the first time in an *in vivo* model the ability of um-PEA to prevent such alterations, thus reducing the abnormal phosphorylation of tau. Besides the deposition of NFTs, it has been demonstrated that a disruption of astrocyte function contributes to neuronal death^39^. In this study, we also investigated the expression of the neuron-specific cytoskeletal marker, MAP2. By immunofluorescence, we show that um-PEA increases MAP2 expression in the CA1 zone of the hippocampus in 3×Tg-AD mice at 6 and 12 months of age, suggesting that um-PEA increases neuronal viability in this region, a key area for the mediation of memory formation and recall.

Alterations in both the glutamatergic system and the metabolism of the brain are well documented in AD patients^45,46^. Employing both *in vivo* MRI/MRS scanning and microdialysis sampling, we evaluated the effects of um-PEA on neural metabolism and glutamatergic transmission in the hippocampus of mice. MRI/MRS revealed that 6-month-old 3×Tg-AD mice show a robust reduction of both Glx and NAA content compared to Non-Tg mice. This observation may underlie the substantial cognitive deficits observed at this age. Interestingly, um-PEA increased Glx content and improved the cognitive performance and behavioral abnormalities observed in 3×Tg-AD mice. Our data also highlight some intriguing potential mechanisms by which AD affects glutaminergic transmission. In younger (6-month-old) 3×Tg-AD mice, we observed a marked increase in the levels of extracellular glutamate, which is probably due to a reduced expression of a glial transporter mainly responsible for glutamate reuptake (GLT-1). Despite observing a similar trend in aging (12-month-old) mice, we did not detect any statistical difference in brain metabolites and extracellular glutamate levels, but a reduction of GLT-1 expression was still evident.

Our data extend the knowledge of the mechanisms underlying the progression of AD-like pathology in 3×Tg-AD mice and demonstrate the exceptional therapeutic potential of um-PEA in restraining the development of AD-like pathology and cognitive/behavioral declines by exerting a combination of anti-inflammatory and neuroprotective effects. Given the numerous and multitargeted effects of um-PEA that we observed, we can speculate that many molecular mechanisms are involved in mediating the actions of this compound. Despite we have already demonstrated, in other preclinical models of AD, that PEA exerts its pharmacological effects through the peroxisome proliferator-activated receptors alpha (PPARα) involvement^11–13,47^, other molecular targets should be taken into account, including PPARγ, transient receptor potential vanilloid type-1 channel, orphan G-protein-coupled receptor 55, and the so-called entourage effect on the endocannabinoid system^19^. Therefore, further experiments will be needed to address this issue. Our results indicate an interesting interaction of um-PEA treatment and the aging process, which may suggest its use as a potential preventive therapy. In aging mice, um-PEA treatment induces an effect on pathways involved in AD pathology, but the most interesting data were derived from younger mice. Indeed, changes in neural function in mice precociously treated with um-PEA (starting from 3 months of age, before any overt signs of disease) seemed to be less pronounced, suggesting the benefits of starting drug treatment at an early stage.

From the perspective of the translational potential of um-PEA in human AD, our data strongly suggest its exceptional potential as a therapy that provides clinically considerable benefits if started early enough in the continuum toward dementia. It is noteworthy that PEA, which is already licensed for use in humans, displays a high tolerability and safety profile and would be an ideal candidate for long-term use lasting several years, as potential AD treatments require.

Summarizing, our data suggest that um-PEA exerts a robust therapeutic effect in a transgenic mouse model (3×Tg-AD) of AD, since it ameliorates both cognitive deficits and a range of neuropathological features. Despite the limits of our preclinical experimental study and avoiding any simplistic extrapolation of data from the animal model to the human condition, the results of this research suggest that um-PEA demonstrates considerable potential to have an impact on the progression of AD.
